# The need for carbon finance schemes to tackle overexploitation of tropical forest wildlife

**DOI:** 10.1111/cobi.14406

**Published:** 2024-10-22

**Authors:** Caroline E. Milson, Jun Ying Lim, Daniel J. Ingram, David P. Edwards

**Affiliations:** ^1^ Ecology and Evolutionary Biology, School of Biosciences University of Sheffield Sheffield UK; ^2^ Durrell Institute of Conservation and Ecology (DICE) University of Kent Canterbury UK; ^3^ Department of Biological Sciences National University of Singapore Singapore; ^4^ Center for Nature‐based Climate Solutions National University of Singapore Singapore; ^5^ Department of Plant Sciences University of Cambridge Cambridge UK; ^6^ Centre for Global Wood Security University of Cambridge Cambridge UK; ^7^ Conservation Research Institute University of Cambridge Cambridge UK

**Keywords:** carbon cycle, climate finance, overexploitation, tropical forests, bosques tropicales, ciclo del carbono, finanzas climáticas, sobreexplotación, 碳循环, 热带森林, 过度利用, 气候金融

## Abstract

Defaunation of tropical forests, particularly from unsustainable hunting, has diminished populations of key seed dispersers for many tree species, driving shifts in forest community composition toward small‐fruited or wind‐dispersed trees with low wood density. Such shifts can reduce aboveground biomass, prompting calls for overexploitation to be included in bioeconomic policy, but a synthesis of existing literature for wildlife impacts on carbon stores is lacking. We evaluated the role of wildlife in tropical forest tree recruitment and found that it was critical to tropical forest carbon dynamics. The emerging financial value of ecosystem services provided by tropical forest fauna highlights the need for carbon‐based payments for ecosystem services schemes to include wildlife protection. We argue for three cost‐effective actions within carbon finance schemes that can facilitate wildlife protection: support land security opportunities for Indigenous peoples and local communities; provide support for local people to protect forest fauna from overexploitation; and focus on natural regeneration in restoration projects. Incorporating defaunation in carbon‐financing schemes more broadly requires an increased duration of carbon projects and an improved understanding of defaunation impacts on carbon stores and ecosystem‐level models. Without urgent action to halt wildlife losses and prevent empty forest syndrome, the crucial role of tropical forests in tackling climate change may be in jeopardy.

## Abstract (es)

Esquemas de financiamiento de carbono necesarios para abordar la sobreexplotación de fauna en los bosques tropicales

**Resumen:**

La defaunación de los bosques tropicales, sobre todo por la caza insostenible, ha disminuido las poblaciones de los principales dispersores de semillas de muchas especies arbóreas, lo que ha provocado cambios en la composición de las comunidades forestales, ahora compuestas por árboles de fruto pequeño o dispersados por el viento y con baja densidad de madera. Estos cambios pueden reducir la biomasa aérea, lo que ha llevado a pedir que la sobreexplotación se incluya en la política bioeconómica, pero no existe una síntesis de la bibliografía existente sobre el impacto de la fauna silvestre en las reservas de carbono. Evaluamos el papel de la fauna silvestre en el reclutamiento de árboles en los bosques tropicales y descubrimos que es fundamental para la dinámica del carbono forestal tropical. El valor financiero emergente de los servicios ambientales prestados por la fauna de los bosques tropicales pone de relieve la necesidad de que los sistemas de pagos por servicios ambientales basados en el carbono incluyan la protección de la fauna silvestre. Argumentamos que actualmente existen tres acciones rentables dentro de los sistemas de financiación del carbono que pueden facilitar la protección de la fauna: garantizar la seguridad de la tenencia a los pueblos indígenas y las comunidades locales; proporcionar apoyo a la población local para proteger la fauna forestal de la sobreexplotación; y centrarse en la regeneración natural en los proyectos de restauración. La inclusión de la defaunación en los planes de financiamiento del carbono de forma más amplia requiere una mayor duración de los proyectos de carbono y una mejor comprensión de los impactos de la defaunación en las reservas de carbono y en los modelos a nivel de ecosistema. Si no se toman medidas urgentes para detener las pérdidas de fauna y prevenir el síndrome del bosque vacío, el papel crucial de los bosques tropicales para hacer frente al cambio climático puede estar en peligro.

## 解决热带森林野生动物过度利用问题的碳金融计划需求

**【摘要】**热带森林的动物群衰退, 尤其是由于不可持续的狩猎, 导致许多树种的关键种子传播者数量减少, 进而推动森林群落组成向小果树或依赖风传播的树种及低木材密度树种转变。这种转变可能会导致地上生物量减少, 这也促使人们呼吁将过度开发利用纳入生物经济政策之中, 但目前仍缺乏有关野生动物对碳储存影响的现有文献综述。我们评估了野生动物在热带雨林树木更替中的作用, 发现其对热带雨林碳动态至关重要。热带雨林动物群所提供的生态系统服务的经济价值正在显现, 这强调了在以碳为基础的生态系统服务付费计划中纳入野生动物保护的必要性。我们认为, 当前的碳金融计划中有三种具有成本效益的行动可推动野生动物保护, 分别是确保土著人民和当地社区的土地权利;为当地人民提供支持, 以保护森林动物免受过度利用;以及在恢复项目中注重自然再生。更广泛地将动物群衰退纳入碳金融计划, 还需要延长碳计划的持续时间, 并深入理解动物群衰退对碳储存及生态系统水平模型的影响。如果不采取紧急措施来阻止野生动物丧失并预防空森林症状的出现, 那么热带森林在应对气候变化方面的关键作用可能会面临严重威胁。**【翻译:胡怡思;审校:聂永刚】**

## INTRODUCTION

Tropical forests store half of Earth's terrestrial carbon (Lewis et al., 2015), but ongoing deforestation and degradation is dramatically reducing their capacity to act as carbon sinks (Pan et al., 2011). Protecting tropical forests and preserving their ability to regenerate and sequester carbon is thus a pivotal part of tackling the climate change crisis.

Animals play a key role in the forest carbon cycle (Schmitz et al., 2018; Sobral et al., 2017), and vertebrates dispersing seeds are crucial in tropical forest dynamics. The majority of tropical rainforest tree species are vertebrate dispersed (Jordano, 2000), a testament to the importance of plant–animal interactions in maintaining tropical tree species diversity. However, pantropically, many vertebrate species are in decline due to unsustainable levels of hunting or overexploitation for subsistence and trade (Benítez‐López et al., 2017, 2019; Morton et al., 2021), resulting in “empty forest syndrome” (Bogoni et al., 2023; Redford, 1992; Wilkie et al., 2011). Low rates of reproduction combined with high hunting pressure make large‐bodied vertebrates acutely sensitive to overexploitation (Bennett & Robinson, 2000), and unsustainable levels of hunting are the primary threat to 301 (26%) of the 1169 terrestrial mammal species listed as threatened with extinction (Critically Endangered, Endangered, or Vulnerable) on the International Union for Conservation of Nature (IUCN) Red List (Ripple et al., 2016). In tropical forests, medium‐ to large‐bodied frugivorous mammals, including orangutans (*Pongo* spp.) and tapirs (*Tapirus* spp.), and birds, such as hornbills (*Bucerotidae* spp.) and cassowaries (*Casuarius* spp.), perform vital seed‐dispersal services for tree species bearing large fruits (Guimarães et al., 2008; Lim et al., 2020), but they are often primary hunting targets (Meijaard et al., 2011; Paula et al., 2022; Peres, 2001; Whytock et al., 2018). Furthermore, other anthropogenic drivers of forest degradation and carbon reduction, such as fragmentation, logging, and roads, exacerbate and enable overexploitation impacts (Edwards et al., 2014; Espinosa et al., 2014; Laurance et al., 2008; Peres, 2001).

Defaunation has downstream consequences for the structure and function of tropical ecosystems, including their ability to store and sequester carbon (Bello et al., 2015; Enquist et al., 2020; Jansen et al., 2010; Osuri et al., 2016; Peres et al., 2016; Young et al., 2016). Key drivers of tropical forest defaunation, from deforestation to fire, are recognized in carbon‐based payments for ecosystem services (CBPES) schemes, including activities reducing emissions from avoided deforestation and degradation (REDD+), and by the Intergovernmental Panel on Climate Change (IPCC). Yet, overexploitation is not recognized (Brodie, 2018; Gardner et al., 2019; Krause & Nielsen, 2019), despite its potential to undermine global efforts to reduce carbon emissions. We evaluated the evidence for defaunation from overexploitation as a driver of significant reductions in carbon stocks across tropical forests, identified core knowledge gaps, and weighed the barriers to and potential for protection against hunting‐induced defaunation in climate finance projects.

## DEFAUNATION IMPACTS ON ABOVEGROUND CARBON STOCKS

### Tree recruitment

Defaunation affects the ecosystem functioning of tropical forests through the alteration of seed dispersal processes. For example, loss or decline in frugivores may lead to reduced seed dispersal and result in increased seed or seedling mortality. This may come from Janzen–Connell effects (Hulsman et al., 2024; Song et al., 2021), a phenomenon in which seeds and seedlings have elevated mortality from natural enemies and intraspecific competition if they are at high densities or close to their mother trees, a situation that may become more likely if seed dispersal is limited by defaunation.

**FIGURE 1:**
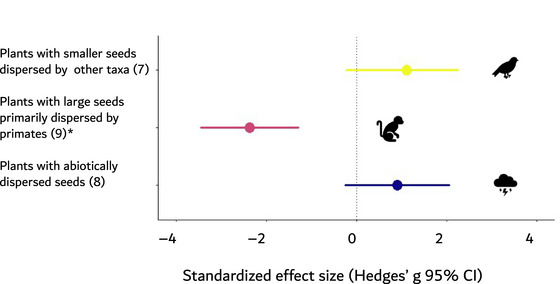
Effect of defaunation on regeneration of woody plants with large seeds and small seeds or that disperse seeds abiotically (numbers in parentheses, number of pair‐wise comparisons between high fauna and low fauna treatments per category; circles, mean standardized effect size [Hedges *g*]; whiskers, 95% confidence intervals; asterisks, categories for which confidence intervals do not overlap zero) (reproduced from Gardner et al. [2019]).

Furthermore, because defaunation is disproportionately greatest among large‐bodied frugivores, which are more likely to disperse large‐seeded tree species (Lim et al., 2020; Young et al., 2016), defaunation may have broader long‐term consequences on forest diversity and composition. A global meta‐analysis of 43 studies, including 19 studies on seed dispersal from across the tropics, revealed that the dispersal of large seeds is negatively affected, whereas small seeds or those dispersed by wind benefit from defaunation (Gardner et al., 2019) (Figure 1). Furthermore, 21 (of 42) studies reviewed by Kurten (2013) investigated seedling distribution, abundance, diversity, survival, or all four factors. Overall, Kurten (2013) found that large‐seeded tree species relying on animals that are hunted to act as dispersal agents are the most negatively affected by defaunation. The limited dispersal of large seeds causes shifts in the community composition of seedlings and juvenile trees toward fast‐growing, pioneer species and those with abiotically dispersed or smaller seeds in forests across the tropics, including Borneo (Harrison et al., 2013), Democratic Republic of Congo (Beaune et al., 2013), and Peru (Nunez‐Iturri et al., 2008).

Simulated extinction of large‐fruited tree species suggests that reductions in the recruitment of large‐fruited species may lead to long‐term reductions in total aboveground biomass (Bello et al., 2015; Chanthorn et al., 2019; Osuri et al., 2016; Peres et al., 2016). In the Brazilian Amazon, defaunation of exploitation‐sensitive frugivores is projected in simulations based on forest plot data of large trees to cause long‐term aboveground biomass reductions up to 37.8%, equating to a pan‐Amazonian loss of 750 million Mg C (Peres et al., 2016). This is in part because many tree species with large seeds have high stemwood density relative to early successional species (Bello et al., 2015; Osuri et al., 2016; Peres et al., 2016) and greater maximum attainable height and basal diameter (Osuri & Sankaran, 2016). A similar simulation model used by Osuri et al. (2016) suggests that the impact of defaunation on aboveground biomass depends on differences in tree assemblage composition, with extinction of the largest seeded trees leading to losses of 2–12% of carbon in African and South American tropical forests.

Yet, the aforementioned simulation models may be overly pessimistic because they are often based on the assumption of complete recruitment failure of large‐seeded species. In Amazonian Peru, seedling community composition was altered in forests used for hunting by migrant workers and thereafter for commercial hunting by Indigenous peoples (IPs). Yet, dispersal and germination of large‐seeded species have occurred since the onset of hunting 32 years prior, resulting in no decline in species dispersed by large, hunted fauna and suggesting effective recruitment into the sapling layer (Hazelwood et al., 2020). Seed dispersal by some remaining faunal species is proffered as a potential explanation for this observation, and this appears vital in the American tropics (Stoner et al., 2007). Secondary seed dispersers, which would normally have moved the seeds beyond their initial site of deposition, may now directly exploit undispersed fruits or seeds under parent trees. In support of this, some large‐fruited species continue to survive despite the extinction of their megafauna dispersers (Guimarães et al., 2008; Lim et al., 2020), probably through suboptimal dispersal by comparatively smaller mammals, such as tapirs, primates, and scatter‐hoarding rodents, and other means, such as gravity and water (Guimarães et al., 2008).

Nevertheless, the loss of tropical American megafauna may have reduced the seed dispersal effectiveness of some large‐fruited tree species by over 95%, potentially causing a 54% reduction in their population (Doughty et al., 2016) and a decline in aboveground biomass and carbon. Rapidly reducing seed size (Galetti et al., 2013) might have enabled some tree species to persist, but it is likely that others relying on megafauna for seed dispersal went extinct (Guimarães et al., 2008; Lim et al., 2020). The loss of 83 species of South American megafauna after the late Pleistocene potentially reduced carbon content in the Amazon by 1.5% (Doughty et al., 2016), despite nearly all of these animals being adapted to open environments (Cione et al., 2009) and thus unlikely to have a significant impact on forest seed dispersal.

The same is not true for the megafauna and other large animals in tropical forests of Africa and Asia, which are only now experiencing their own devastating losses—at a much faster rate than the historical tropical American megafaunal loss (Barnosky et al., 2011). Reduced seed dispersal, especially for obligately dispersed plant species, for example, by chimpanzees (*Pan troglodytes*) (Wrangham et al., 1994), African forest elephants (*Loxodonta cyclotis*) (Beaune et al., 2013), and Sumatran rhino (*Dicerorhinus sumatrensis*) (McConkey et al., 2022), and the inability of many species to reduce seed size or rely on remaining or secondary dispersers could see overwhelming reductions in the abundance and range of large fleshy‐fruited tree species (Lim et al., 2020). This is likely to be on a far larger scale than following South America's megafaunal extinctions because habitat loss and degradation, in addition to overexploitation, are concurrently driving population declines of hundreds of medium‐ to large‐bodied seed‐dispersing species (Benítez‐López et al., 2019; Morton et al., 2021). The long‐term consequences for tropical forest tree communities and carbon storage could be immense.

### Trophic cascades

**FIGURE 2:**
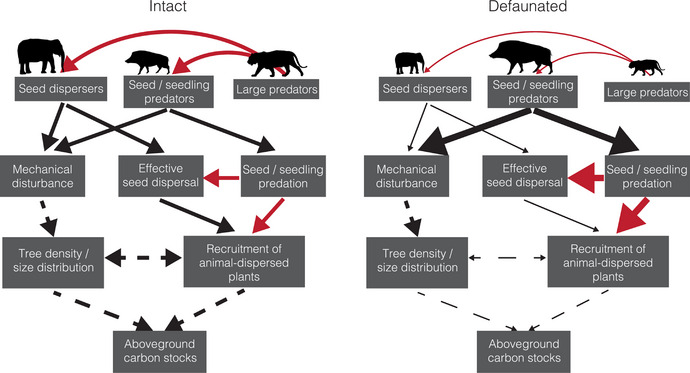
Impacts of defaunation on animal functional groups, ecological processes, and aboveground carbon stocks (red arrows, negative effects; black arrows, positive effects; dashed lines, effects that are less well understood; large icon size, population increase; small icon size, population decrease; thick arrows, defaunation increases a particular process or relationship; thin arrows, defaunation decreases process or relationship).

The effects of defaunation extend beyond the direct impacts of reduced seed dispersal and tree recruitment. Foraging by African forest elephants reduces tree recruitment in Gabonese forests but is also associated with low liana infestation (Terborgh et al., 2016), whereas in the Republic of Congo, aboveground biomass and carbon are enhanced by forest elephant disturbance (Berzaghi et al., 2023, 2019) (Figure 2). It is predicted that up to 96% of Central African forests will have modified species composition and structure if the extirpation of forest elephants from most of their historical range continues; recruitment of large trees and carbon stocks will be reduced (Poulsen et al., 2018). The interactions between large herbivores and tropical forest vegetation are complex, but overall these animals have positive climate mitigation and adaptation impacts on vegetation structure and carbon stock above‐ and belowground (Berzaghi et al., 2023, 2019; Malhi et al., 2022).

Carnivores also play a positive role in the carbon cycle (Malhi et al., 2022). Some species, such as civets (Viverridae) and otters (e.g., *Aonyx* spp.), perform vital seed dispersal services (Stoner et al., 2007), and the loss of predators can trigger trophic cascades, which negatively affects forest dynamics, including plant communities and carbon sequestration (Estes et al., 2011; Schmitz et al., 2018; Sobral et al., 2017) (Figure 2). The near absence of predators and frugivores on small islands in Venezuela saw a dramatic reduction in seedlings and saplings of canopy trees compared with the mainland (Terborgh et al., 2001). An abnormally high population of wild pigs (*Sus scrofa*) (supported by nearby oil palm) in a primary lowland forest in Malaysia saw a 62% decline in forest tree sapling density over the 24‐year study period (Luskin et al., 2017). Excluding the pigs from study plots saw an 83% increase in tree seedling abundance (Luskin et al., 2019). This reflects the increased mechanical disturbance (Luskin et al., 2019) and sapling removal for nests (Luskin et al., 2017) by the pigs, but these effects could be common in other tropical forests depleted of natural predators. Large herbivores, and in some places, carnivores, are often specifically targeted by hunters or caught in indiscriminate snare traps, and when these animals are absent, the top‐down controls they provide are also lost, which has negative consequences for forest carbon (Berzaghi et al., 2019; Estes et al., 2011; Malhi et al., 2022; Poulsen et al., 2018; Terborgh et al., 2016).

The role of insectivorous species in regulating vegetation communities in tropical forests is unknown, but the diet of many hunted species, including pangolins (Manidae), tamanduas (Myrmecophagidae), armadillos (Cingulata), and sun bears (*Helarctos malayanus*), is primarily ants (Formicidae) and termites (Termitoidae). Although termites have important ecological roles in tropical forests, infestation can have negative impacts on tree health and mortality (Gely et al., 2021). In drought conditions, termite abundance doubled in Bornean forest, accelerating ecosystem processes and increasing liana seedling survival (Ashton et al., 2019), thereby providing a potential competitive advantage over large‐fruited tree species and subsequent consequences for forest aboveground biomass and carbon sequestration.

## DEFAUNATION AND CLIMATE FINANCE

Defaunation as a result of overexploitation has great potential to erode carbon sequestration, substantially alter the carbon cycle, and undermine climate change initiatives (Brodie & Gibbs, 2009; Doughty et al., 2016; Gardner et al., 2019; Malhi et al., 2022; Osuri et al., 2016; Peres et al., 2016). In financial terms, the mutualism between large frugivores and woody tree species in the Brazilian Amazon is valued at up to US$13.65 trillion (Peres et al., 2016). In Brazil's Atlantic Forest (Bello et al., 2021), seed dispersal services are valued from US$3.94 to US$15.42 ha^−1^ year^−1^, equivalent to US$39–154 million per year across the remaining Atlantic Forest. Carbon capture by African forest elephants is valued at US$20 billion over the next 10 years if the remaining populations are protected; their extinction over 10–30 years would result in US$2–7 billion of lost carbon services (Berzaghi et al., 2022).

These ecological and financial impacts have prompted calls for CBPES, including REDD+, to acknowledge and tackle unsustainable hunting and wildlife trade (Bello et al., 2015; Gardner et al., 2019; Krause & Nielsen, 2019). In theory, by paying to remove or manage threats, CBPES protect carbon in the long term, promote sustainable management of forests, and conserve biodiversity. Yet, currently, defaunation threatens to undermine the financial integrity, carbon storage potential, and biodiversity targets of many CBPES schemes. Carbon‐based payments for ecosystem services can address overexploitation and ensure carbon storage is maximized for little or no additional financial outlay by securing land tenure for IPs, supporting local communities (LCs) in managing hunting sustainably, and increasing emphasis on natural regeneration in restoration projects.

### Support land security opportunities for IPs and LCs

Indigenous lands are critical for conservation and sustainable development agendas, covering 25% of Earth's land surface (Garnett et al., 2018) and containing 41% of IUCN‐assessed threatened terrestrial mammals (O'Bryan et al., 2021). Deforestation and degradation rates on Indigenous land across the tropics already compare favorably with those in protected areas (Sze et al., 2022), and wildlife offtake managed by Indigenous communities can be sustainable (Dawson et al., 2021). Yet, some Indigenous lands face increasing unsustainable human pressure, often from external forces seeking to exploit forest resources (Dawson et al., 2021; O'Bryan et al., 2021), which exposes Indigenous environmental defenders to criminalization, violence, and assassinations (Scheidel et al., 2020)

Securing tenure rights of IPs and LCs can encourage local environmental stewardship and lead to positive social and ecological outcomes (Dawson et al., 2021). The IPCC recognizes this and has appealed for actions to strengthen land access and tenure security for IPs and LCs (IPCC, 2022). An additional $1.7 billion in climate finance was pledged at COP26 specifically to secure the land rights of IPs and LCs. Yet, granting formal legal titles is only one small part of land tenure security. Legal and institutional recognition, empowerment, respect, and support for the rights of IPs and LCs (particularly women), positive stakeholder relations, and the ability to engage in sustainable livelihoods are often equally important (Dawson et al., 2021; Larson et al., 2023).

As the land of IPs and LCs is often in protected areas or major global carbon sinks (Garnett et al., 2018; Haenssgen et al., 2022), there is potential for conflict with conservation goals and restrictions. State‐led, top‐down actions designed to protect forest resources on Indigenous lands often undermine conservation effectiveness (Haenssgen et al., 2022; Obura et al., 2021). Climate finance could instead promote and fund the use of intermediary mechanisms, such as the Tenure Facility or the Peoples Forest Partnership. These projects can facilitate the delivery of outcomes that benefit IPs and LCs, forest carbon, and biodiversity, while also enabling forest communities to engage with climate finance. The presence of nongovernmental organizations (NGOs) can significantly increase the probability of deforestation policy adoption and permanence by IPs and LCs (Tanner & Ratzke, 2022) and can act as independent witness to encroachment by external actors. Confirmation by NGOs of encroachment can then corroborate the need for effective support and protection for IPs and LCs defending their ancestral lands from outside forces, such as industrial logging and market‐based extraction of wildlife.

### Reduce or manage hunting in existing projects

Animals are hunted pan‐tropically for subsistence, as a source of income, and for traditional and sociocultural purposes (Ingram et al., 2021). The need for sustainable use is increasingly acknowledged in global policy (Ingram et al., 2021; IPBES, 2022) and has been prioritized as one of the main goals in the Kunming–Montreal Global Biodiversity Framework (CBD, 2022; Obura et al., 2023). Yet, financing support for LCs to achieve sustainable use at scale remains a significant challenge (Ingram et al., 2021).

Where tropical forests face threats from anthropogenic activities, IPs and LCs can be given support to reduce overexploitation through several means. For example, climate finance could be used for LCs to engage in activities that can reduce overexploitation, illegal logging, and clearance for agriculture, bringing multiple benefits for forest carbon. Where hunting is locally important and legal, climate finance could support LCs in managing hunting within sustainable limits, including through wildlife monitoring and species‐specific sustainability assessments. If hunting is illegal or is only legal for a subset of people (such as IPs), assuming that in‐country laws are appropriate and ethical (van Vliet et al., 2019), local people could be employed or supported to employ others to patrol forests. This may deter external groups that are not local wildlife stewards from exploiting forest resources and generate livelihoods in places where hunting may be one of the only sources of income. Such community‐based systems can have positive outcomes for people and wildlife (Brunner et al., 1999).

Carbon‐based payments for ecosystem services programs vary in scale from national‐ to local‐level actions, but engaging and not marginalizing IPs and LCs is critical for social and environmental success (Bennett & Robinson, 2000; Dawson et al., 2021; Sze et al., 2022). Given the complex and highly variable socioecological dynamics of hunting within communities, their active involvement in the design, decision‐making, and monitoring of interventions to reduce unsustainable hunting pressures is critical, ideally with support from government agencies, scientists, and NGOs to achieve hunting sustainability (Robinson & Bodmer, 1999). Projects should support Indigenous and other forest‐dwelling communities to identify risks, only proceed with vetted interventions, and use conditions and sanctions identified, agreed on, and enforced by local people. Ongoing evaluation and monitoring are advisable to avoid climate‐finance‐funded activities becoming simply an additional income, rather than reducing overexploitation (Brown, 2003; Roe et al., 2015).

### Forest restoration

Countries across the tropics have pledged to restore millions of hectares of deforested landscapes under the Bonn Challenge at a cost of up to $34,000 per hectare (Crouzeilles et al., 2017), but wildlife presence is key in maximizing forest restoration success (Gardner et al., 2019; McAlpine et al., 2016; Parrotta & Knowles, 2001). For example, seed dispersal by bats and birds was responsible for 94% of animal‐dispersed tree and shrub species in one restoration plot in abandoned pasture in southern Mexico over just 6 years (Peña‐Domene et al., 2014). In contrast, the absence of vertebrate seed dispersers in regenerating forest on Guam reduced tree species richness by 50% compared with neighboring islands with intact faunal communities (Wandrag et al., 2017). The absence of wildlife in many tropical forest restoration projects is likely to hinder successional processes (Wunderle, 1997) and ultimately diminish carbon sequestration potential.

Natural and assisted regeneration of tropical forests outperforms active restoration for biodiversity and vegetation structure (Crouzeilles et al., 2017) and could reduce planting costs by US$90 billion in the Brazilian Atlantic Forest alone (Crouzeilles et al., 2020). Embracing natural regeneration would provide significant financial savings, which could fund wildlife protection in existing adjacent forest where hunting pressure is high (Parry et al., 2009). The successful restoration of a disused bauxite mine in the Brazilian Amazon was partly due to an effective ban on hunting; approximately 75 tree species are present as a result of vertebrate seed dispersal (Parrotta & Knowles, 2001).

Projects seeking to connect old‐growth forest fragments, such as the Atlantic Forest Restoration Pact, could realize multiple carbon sequestration benefits by protecting fauna from overexploitation. Dispersal of large‐seeded tree species in fragments is dramatically reduced from the levels observed in contiguous forest due to the lower abundance of large‐bodied dispersers present (Cordeiro & Howe, 2001; Cramer et al., 2007). Reconnecting forest fragments and protecting the extant wildlife in fragments and adjacent continuous forest would safeguard seed dispersal in existing forest stands, restore seed dispersal functioning in fragments, and optimize regeneration outcomes in restoration sites. In the Brazilian Pantanal, endangered wildlife threatened by hunting used restored forest corridors connecting fragments to protected state parks within a decade (Chazdon et al., 2020), demonstrating that the benefits of reconnecting forest fragments can be realized quickly. As with CBPES projects tackling overexploitation, engagement with IPs and LCs is key (Bennett & Robinson, 2000; Sze et al., 2022).

### Barriers to wider implementation of defaunation in CBPES

The impacts of overexploitation on aboveground carbon stocks in tropical forests will likely play out over long periods (Doughty et al., 2016; Peres et al., 2016). Because the largest trees contain the vast majority of aboveground carbon biomass (Lutz et al., 2018), it may be many years before changes in recruitment dynamics precipitated by faunal losses translate into substantial impacts on forest composition (Harrison et al., 2013). As such, most studies evaluating the effect of defaunation have relied on simulations (Bello et al., 2021, 2015; Chanthorn et al., 2019; Osuri et al., 2016; Peres et al., 2016). Typically, such simulations are zero‐sum models of tree removal and replacement applied to observed forest plots. Tree species whose recruitment are hypothesized to be most affected by defaunation are incrementally or completely removed and replaced by random individuals drawn from the remaining community, whereas either basal area or stem density of the original forest plot is maintained. Because these models do not require data on rates of recruitment or mortality (or on how they may be affected by overexploitation), they can be broadly applied to forest plot data, but their methodological simplicity may hinder their application in carbon valuation schemes.

Regional differences in tree assemblage composition may also generate highly variable impacts of defaunation on carbon storage across tropical forests. For example, in the aseasonal tropics of Southeast Asia, hardwood wind‐dispersed Dipterocarpaceae species dominate, so reductions in the recruitment of animal‐dispersed trees may only have a modest impact on aboveground carbon stocks (Osuri et al., 2016), but this is contested (Chanthorn et al., 2019). Temporally explicit, ecosystem‐level models (Berzaghi et al., 2019) are more demanding to parameterize and require a good understanding of defaunation impacts on forest ecosystem functioning but can generate accurate projections of carbon change. The intersection of vegetation composition, dispersal modes, and wood density must inform individual‐based models to predict carbon loss across an explicit time frame at local and regional levels. These predictions can then inform more accurate and measurable outcomes for tree community composition, carbon storage, and biodiversity so that whether CBPES targets reducing overexploitation impacts are met in a realistic time frame can be assessed.

The short duration of many CBPES projects is a further challenge to including targets preventing or reversing defaunation. Large animals have low reproduction rates (Fenchel, 1974), and it may be several generations before the success of hunting management becomes apparent, long past the time frame of current CBPES programs. In turn, because improved seed dispersal relies on increased abundance of large vertebrates, it too is difficult to measure in the short term.

These difficulties in measuring carbon gains and the short shelf life of many carbon finance projects could make diverting resources to halt and reverse defaunation unattractive to funders and policy makers, especially given the alternatives of using climate finance to avoid deforestation and logging or increase sequestration via forest restoration. However, the emerging financial value of ecosystem services provided by tropical forest fauna (Bello et al., 2021; Berzaghi et al., 2022; Peres et al., 2016) highlights the need for CBPES to place more emphasis on safeguarding wildlife populations, increase project timescales, and ensure extra finance is available to address the overexploitation threat to carbon stores. In addition to the substantial savings to the climate finance budget from embracing natural regeneration, the integration of climate finance projects in projects of organizations that have similar goals, such as the Convention on Biological Diversity, IUCN, and conservation organizations, could achieve enormous cost savings (Gardner et al., 2012). LCs can collect forest data of comparable quality to scientists at half the cost, and such engagement would empower IPs and LCs to own and monitor carbon stocks while contributing to local livelihoods and biodiversity conservation (Danielsen et al., 2013, 2011). If the irreplaceable role of animals in the carbon cycle were integrated into global financial markets (Berzaghi et al., 2022), this would provide additional funds for CBPES projects to protect wildlife from unsustainable hunting.

## FUTURE DIRECTIONS AND CONCLUSIONS

Although there is a significant, growing body of evidence that the overexploitation of wildlife negatively affects tropical forest regeneration, the extent to which reduced seed dispersal, increased seed and seedling predation, and overabundance of lianas drive these changes remains unclear. Understanding the importance of different trophic guilds in maintaining tree species composition and carbon sequestration would be improved by further study, and evidence clarifying whether forest regeneration across the tropics is variable due to historic extinctions, secondary dispersal, local‐level forest composition, and nonredundant dispersers remains absent.

These ecological links are difficult to quantify but need urgent clarification and integration into more powerful, temporally explicit, and mechanistic models, especially because effects of defaunation on forest functioning may take decades to centuries to play out and detect. Ecosystem demographic models (Berzhagi et al., 2019) and individual‐ or agent‐based models (Dantas de Paula et al., 2018) may be promising tools for this endeavor.

Despite current limitations, potential exists for incorporating actions that tackle overexploitation in some CBPES programs. When such actions support LCs, programs could protect tropical forest and biodiversity longer, maximize carbon sequestration in regenerating forest, and increase understanding of ecological dynamics in forest recovering from defaunation. This knowledge is key for accurate assessment of carbon loss in tropical forest but increased focus on tackling overexploitation must be the priority, alongside reducing demand for tropical forest wildlife products. Avoiding—or reversing—the empty forest syndrome will eliminate the threat overexploitation poses to carbon sequestration. If climate change initiatives are to continue embracing the irreplaceable nature‐based solutions provided by tropical forest, climate finance must contribute to safeguarding nature's engineers: wildlife.
